# Ornamental Vascular Plant Diversity in Basilicata (Southern Italy)

**DOI:** 10.3390/plants14213306

**Published:** 2025-10-29

**Authors:** Emilio Di Gristina, Raimondo Pardi, Fortunato Cirlincione, Giuseppe Venturella, Maria Letizia Gargano

**Affiliations:** 1 Department of Agricultural, Food and Forest Sciences, University of Palermo, Viale delle Scienze Bldg. 5, 90128 Palermo, Italy; emilio.digristina@unipa.it (E.D.G.); giuseppe.venturella@unipa.it (G.V.); 2 NBFC—National Biodiversity Future Centre, 90133 Palermo, Italy; 3 Department of Soil, Plant and Food Science (Di.S.S.P.A.), University of Bari “Aldo Moro”, Campus “E. Quagliarello”, Via Edoardo Orabona 4, 70125 Bari, Italy; marialetizia.gargano@uniba.it

**Keywords:** alien species, monumental ornamental trees, parks, private gardens, street trees, urban biodiversity

## Abstract

This investigation focuses on urban ornamental greenery, a field of research that is still relatively unexplored in Italy but is becoming increasingly important both from a botanical point of view and in relation to sustainable land management and planning. A checklist of the ornamental vascular flora of Basilicata (Southern Italy) is reported here. A total of 281 taxa were recorded, including trees, shrubs, herbaceous plants, and succulents cultivated in parks, gardens, and street trees. Such taxa (including 265 species s. str., 6 varieties, 5 subspecies, and 11 forms) belong to 201 genera, included in 94 families, among which the most represented are Rosaceae, Oleaceae, Asteraceae, Pinaceae, Cupressaceae, and Fabaceae. Phanerophytes represent the dominant growth form, and the chorological spectrum is composed mainly of Asian and American taxa. Taxa from subtropical and tropical biomes also showed a significant presence. This study highlighted the clear prevalence in the Basilicata ornamental flora of alien taxa (approximately 80%, of which 21% are naturalized aliens) compared to native ones, which is a phenomenon that is unfortunately widespread and observed worldwide.

## 1. Introduction

The term “ornamental plants” usually refers to plants cultivated for aesthetic and decorative purposes [1]. These plants are defined as ornamental for their characteristics, such as the beauty of their flowers and leaves, their pleasant fragrance, and the attractive texture of their foliage, which motivate their cultivation [2,3,4].

From an aesthetic perspective, ornamental plants contribute to the beauty of the landscape and are commonly used in the planning of lawns, gardens, shopping centers, and landscaped areas [5]. They are intentionally cultivated for decorative purposes rather than for food production or by-products, and can be employed as architectural elements, in flower beds, hedges, or on sunny windowsills.

Beyond their visual appeal, many ornamental species are also distinguished by their pleasant fragrance, which enhances their overall aesthetic value [2].

However, their role goes far beyond a mere decorative function: they are fundamental components of the urban environment, capable of providing numerous essential ecosystem services. These include climate regulation through shading and evapotranspiration, which help mitigate the effects of urban heat islands; air purification through the absorption of gaseous pollutants and particulate matter; and the ability to retain and filter storm water, reducing the risks of soil erosion and flooding, as well as improving the quality of wastewater [6,7]. They also contribute to nutrient cycling and the evolution of fertile soils, support crucial ecological processes such as pollination, and provide food and shelter for urban wildlife, particularly pollinating insects and birds [8]. Added to these are cultural, psychological, and social benefits: green spaces enriched with ornamental plants promote citizens’ psycho-physical well-being, stimulate social interaction, offer recreational opportunities, and serve as inspiration for artistic and creative activities [9].

At the same time, the critical aspects associated with the use of ornamental species cannot be overlooked. Many taxa are known for their toxic properties due to the presence of alkaloids, cyanogenic glycosides, saponins, or other bioactive molecules, which can cause serious adverse effects if accidentally ingested by children or pets [10]. Other species release allergenic substances in the form of pollen, latex, or volatile compounds, posing problems for sensitive individuals [11]. In this context, a study recently conducted in Sicily highlighted that most ornamental species cultivated in urban parks and gardens present potential toxic or allergenic risks, emphasizing the need for stricter criteria in their selection and management [12]. Another critical element is the role of ornamental species as vectors for the introduction of alien invasive plants. The extensive global trade of ornamental plants facilitates the spread of exotic taxa, many of which show strong invasive potential, with significant consequences for native ecosystems, local biodiversity, and the ecological functions of urban and periurban habitats [13,14].

Despite the ecological, social, and economic importance of the ornamental sector, scientific research dedicated to these species remains limited, particularly in Italy, where specific studies are sporadic and mostly included in broader investigations on alien vascular flora or invasive species [15,16]. In contrast, in other countries, ornamental plants have been the subject of in-depth research highlighting their multifunctionality and potential applications. Some studies have tested their effectiveness in phytoremediation processes, demonstrating the ability of different ornamental species to tolerate and accumulate heavy metals, thereby enabling soil and water decontamination interventions [17,18]. Other investigations have analyzed the use of ornamental plants in constructed wetlands for the treatment of urban and industrial wastewater, showing that the combined use of multiple species can improve purification performance while reducing environmental impact [19]. Many other studies have documented the ability of certain ornamental species to remove volatile organic compounds (VOCs) and other harmful substances from the air, benefiting indoor air quality and occupant health [20]. Finally, some ornamental species have been evaluated as sensitive bioindicators of air or soil pollution, representing useful tools for environmental monitoring, while other studies have explored the toxicity of shrubs and trees widely cultivated for aesthetic purposes but potentially hazardous to humans and urban fauna [21,22].

In recent years, however, systematic studies of ornamental flora have also progressed in Italy. In particular, two recent publications have provided specific and comprehensive contributions regarding the Apulia [23] and Sicily [24] regions. In Apulia, 287 ornamental taxa were recorded, whereas in Sicily, where the study of ornamental flora has historical roots going back more than forty years and can be considered the best-documented region in Italy on this topic, 928 taxa were recorded. In both studies, a significant percentage of taxa were included in the list of Italy’s alien vascular flora, with a strong predominance of occasional aliens and naturalized neophytes. These results highlight, on the one hand, the extraordinary floristic richness associated with ornamental use in the two regions, and on the other, the need to develop further research aimed at systematizing knowledge at the national level, while promoting sustainable management strategies and preventing the risks associated with the spread of potentially invasive species.

**Figure 1:**
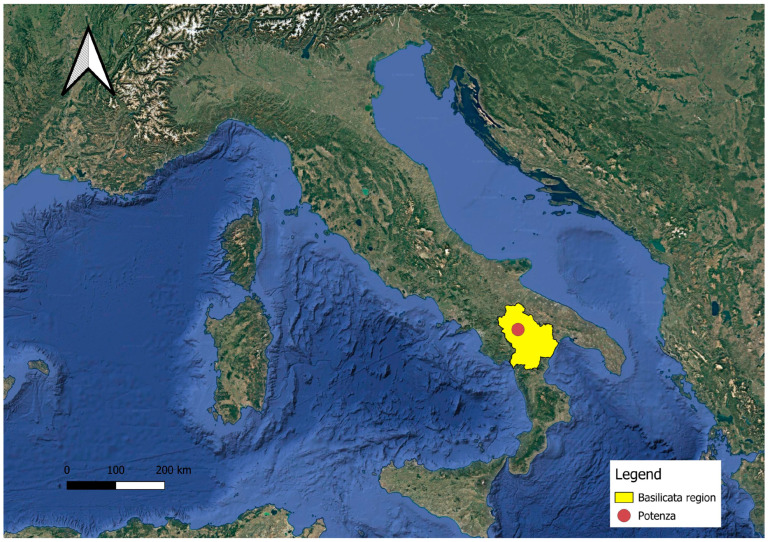
Geographical location of the Basilicata region.

In this survey, we report the first contribution to the checklist of the ornamental vascular flora of the Basilicata region (Southern Italy) (Figure 1). Basilicata, with an area of approximately 9995 km^2^, is among the smallest regions in Italy and, with just over half a million inhabitants, has one of the lowest population densities in the country [25]. The administrative structure is divided into only two provinces: Potenza, which serves as the regional capital, and Matera. Its territory functions as a geo-crossroads between the Adriatic side (Apulia), the Tyrrhenian side (Calabria), and the inland area (Campania), with outlets to both the Tyrrhenian and Ionian Seas. The dominance of the Lucanian Apennines results in a predominantly mountainous and hilly topography, interrupted by a few plains [25]. This orographic structure, with altitudinal ranges from sea level up to 2267 m at Monte Pollino, creates, despite Basilicata’s limited territorial extent, a complex climatic mosaic: the inland areas exhibit a continental climate with cold, snowy winters; the hilly zones show a temperate sub-continental climate; along the Ionian coast there is a hot-arid Mediterranean alternation; the smaller Tyrrhenian strip enjoys milder and more humid conditions [25]. This climatic complexity allows the region to host a wide spectrum of plant taxa from different bioclimatic areas. Currently, knowledge of the ornamental flora of Basilicata is almost entirely lacking. Indeed, no specific contributions are available, and the only information comes from studies that briefly report the naturalization status of some cultivated exotic species in the region [26].

Our investigation, although preliminary, aims to fill this gap by compiling a checklist of the main ornamental taxa cultivated in the Basilicata region. For the purposes of this study, the term “ornamental plants” is used in its broadest sense, encompassing both native and non-native taxa, including trees, shrubs, annual and perennial herbaceous species, as well as bulbs and tubers, all cultivated for decorative purposes in street tree plantings, historic villas, and public and private gardens in the Basilicata region. In addition to its descriptive purpose, the study aims to provide a knowledge base useful for subsequent evaluations by local administrations, both in terms of the aesthetic and landscape enhancement of the species employed and for the analysis of potential risks associated with their spread. Particular attention is devoted, in this context, to issues related to the invasive capacity of certain species and their effects on public health, especially with regard to the possible increase in allergies.

## 2. Results

**Figure 2:**
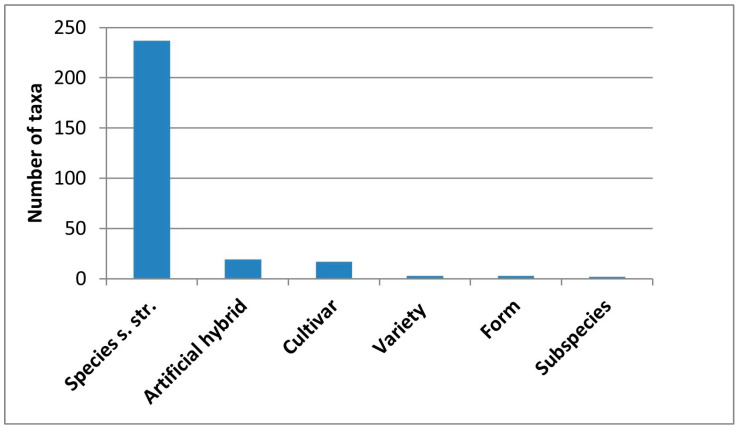
Taxonomic ranks of the ornamental taxa of Basilicata.

A total of 281 taxa were recorded (Appendix A Table A1), distributed as follows: 237 species sensu stricto, 2 subspecies, 3 varieties, 3 forms, 17 cultivars, and 19 hybrids (Figure 2). Specifically, 1 taxon belongs to Pteridophyta, 1 to Ginkgophyta, 1 to Cycadophyta, 29 to Pinophyta, and 249 to Magnoliophyta (of which 214 are Magnoliopsida and 35 Liliopsida). The recorded taxa belong to 201 genera, distributed in 94 families.

**Figure 3:**
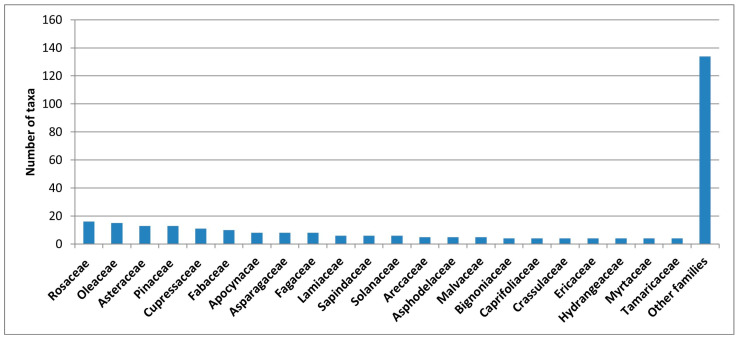
Number of taxa per families in the ornamental flora of Basilicata.

The families with the highest number of specific and infraspecific taxa are Rosaceae (16 taxa, 5.7%), Oleaceae (15 taxa, 5.3%), Asteraceae (13 taxa, 4.6%), Pinaceae (13 taxa, 4.6%), Cupressaceae (11 taxa, 3.9%), and Fabaceae (10 taxa, 3.6%) (Figure 3). The genera with the highest number of taxa are *Ligustrum* (7), *Quercus* (6), *Acer* (5), *Abies* (4), *Tamarix* (4), and *Viburnum* (4).

**Figure 4:**
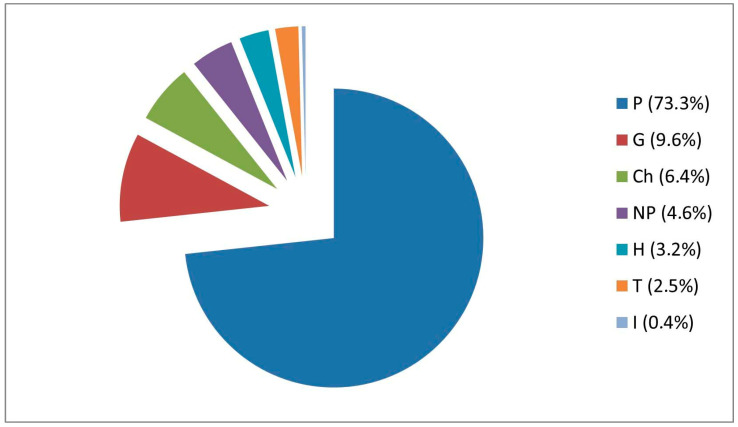
Spectrum of growth forms of ornamental taxa in Basilicata classified according to [27,28] (P, Phanerophyte; NP, Nanophanerophyte; Ch, Chamaephyte; H, Hemicryptophyte; G, Geophyte; T, Therophyte; I, Hydrophyte).

Regarding growth forms, there is a predominance of phanerophytes (P) (particularly scapose and cespitose), i.e., 206 taxa. They are followed by geophytes (G) (27 taxa), chamaephytes (Ch) (18 taxa), nanophanerophytes (NP) (13 taxa), and hemicryptophytes (H) (9 taxa). Lower percentages are observed in therophytes (T) (7 taxa) and hydrophytes (I) (1 taxon) (Figure 4).

**Figure 5:**
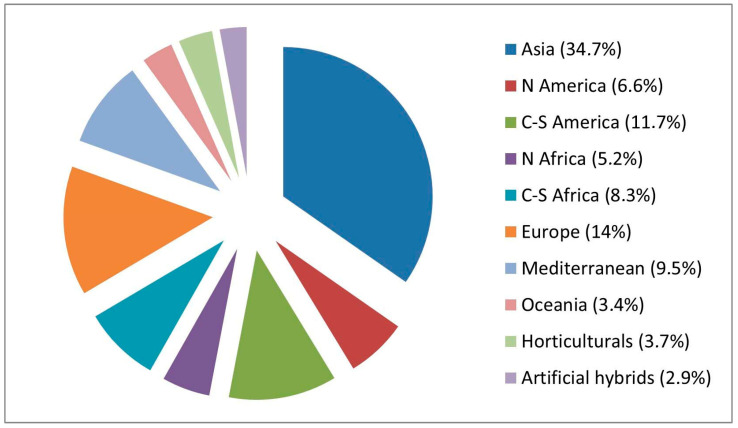
Percentage of taxa by geographical origin (according to [27]).

Regarding the geographic origin of the recorded taxa, the largest percentage is represented by the Asian contingent, followed by the American contingent and the European one. The African contingent represents the dominant component, with the Mediterranean and Oceanic groups following in relative abundance. Horticultural taxa and artificial hybrids are comparatively minor elements within the dataset (Figure 5).

The most represented biome of origin is the temperate biome (160 taxa, 57.0%), and the vast majority of taxa, with respect to their residence time in Italy, fall into the neophyte category (135 taxa, 48.0%).

Regarding their status in Italy, only 64 taxa are native, while 215 taxa are alien; the remaining taxa belong to the historical category (1 taxon) and the cryptogenic category (1 taxon).

**Figure 6:**
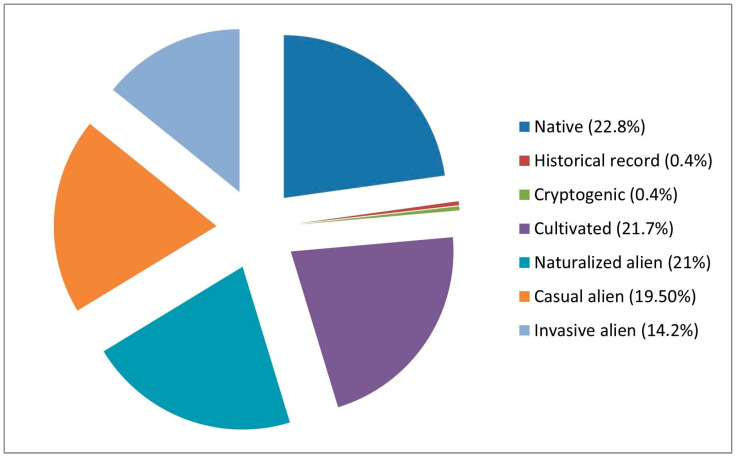
Status in Italy (according to [26,29]) and percentage of ornamental taxa.

Considering the tendency of alien taxa to naturalize, 61 taxa are cultivated, 59 taxa are naturalized (i.e., tending to form stable populations), 55 taxa are casual aliens (i.e., showing a tendency to naturalize but not forming stable populations separate from cultivated plants), and 40 taxa are invasive aliens (i.e., potentially posing a threat to biodiversity by competing with native species) (Figure 6).

## 3. Discussion

The census carried out within the framework of our research has made permitted to draw up a preliminarylist of ornamental taxa from Basilicata (Appendix A Table A1), highlighting how the bioclimatic heterogeneity of the region, which extends from coastal areas to hills and up to the mountainous reliefs of the Lucanian Apennines, creates ecological conditions suitable for the cultivation of ornamental species originating from different geographical and climatic contexts. In fact, in mountainous or hilly areas, taxa typical of cold and temperate climates are found, such as *Abies alba* Mill., *A. cephalonica* Loudon, *A. pinsapo* Boiss., *Cedrus atlantica* (Endl.) Manetti ex Carrière, *C. deodara* (Roxb. ex D. Don) G. Don, *C. libani* A. Rich., *Picea abies* (L.) H. Karst., *P. pungens* Engelm., *Sequoia sempervirens* (D. Don) Endl., *Taxus baccata* L., etc. Along the coasts, the warm climate allows the establishment of species of subtropical and tropical origin, such as *Aeonium arboreum* (L.) Webb & Berthel., *Agapanthus africanus* (L.) Hoffmanns., *Araucaria columnaris* (G.Forst.) Hook., *A. heterophylla* (Salisb.) Franco, *Cascabela thevetia* (L.) Lippold, *Hibiscus × rosa-sinensis* L., *Metrosideros excelsa* Sol. ex Gaertn., *Musa × paradisiaca* L., *Syagrus romanzoffiana* (Cham.) Glassman, etc. Although the coasts cover a limited area compared to the rest of the region, when subtropical and tropical species are considered together, they account for almost 40% of the recorded taxa, a significant percentage that testifies to the importance of this contingent in characterizing the regional ornamental flora of Basilicata. The contingent of Mediterranean climate taxa also proved to be well represented overall, bearing witness to the growing interest in the use of native species for ornamental purposes and, more generally, to greater attention towards sustainable solutions compatible with local ecological conditions.

This heritage intertwines with another element of great importance in the Basilicata plant landscape, namely the presence of monumental species and so-called monumental trees which, while largely belonging to native taxa such as *Castanea sativa* Mill., *Fagus sylvatica* L., *Quercus cerris* L., *Q. pubescens* Willd., *Pinus heldreichii* subsp. *leucodermis* (Antoine) A.E.Murray, and *Taxus baccata*, also include species introduced for ornamental purposes and now fully integrated into the regional landscape. A striking example is represented by *Sequoia sempervirens* cultivated in Campomaggiore Vecchio (Potenza) [30,31]. The coexistence of exotic ornamental species and native monumental trees prompts reflections on the importance of reconciling aesthetic enhancement with the conservation of genetic resources and historical-cultural heritage. From this perspective, urban green planning and management strategies should be oriented towards resilient taxa adaptable to Basilicata bioclimatic conditions and ongoing climate change, while simultaneously promoting the protection of centuries-old specimens, recognized as elements of high ecological, identity, and landscape value.

The census of ornamental flora in Basilicata has also highlighted a dual aspect of relevance for urban and periurban green planning: on the one hand, the presence of many taxa that combine aesthetic value with productive function; on the other, the spread of ornamental species that can have negative effects on human health, both due to intrinsic toxicity and allergenic potential. The use of ornamental plants that also play a role in providing food represents a strategic resource from the perspective of landscape multifunctionality. Species such as *Castanea sativa*, *Citrus × aurantium* L., *C. × limon* (L.) Osbeck, *Corylus avellana* L., *Diospyros kaki* Thunb., *Juglans regia* L., *Musa × paradisiaca* L., and *Punica granatum* L. not only enrich green spaces with their aesthetic value and the seasonality of their blooms but also provide food products of high nutritional and cultural value. These plants, rooted in the Mediterranean agricultural and culinary tradition, also represent a bridge between the botanical and agro-food heritage of the region, contributing to strengthening the sense of local identity and promoting sustainable green management practices. Species such as *Aloe arborescens* Mill. and *A. vera* L. add further value as they offer phytotherapeutic and medicinal benefits, thus acting as ornamental plants with a functional role in promoting well-being [32,33].

At the same time, our study highlighted the need for greater awareness regarding the use of potentially harmful ornamental taxa. Some genera and species very common in urban contexts, such as *Cupressus* spp., *Hesperocyparis* spp., *Pinus* spp., *Ailanthus altissima*, *Olea europaea* L., *Quercus ilex* L., and *Populus* sp. pl., are responsible for allergic phenomena such as pollinosis [34,35,36], which represent an increasing problem for public health, especially in densely populated areas and near schools and hospitals. Another negative aspect is the presence of poisonous ornamental plants such as *Cascabela thevetia* (L.) Lippold, *Laburnum anagyroides* Medik., *Melia azedarach* L., *Nerium oleander* L., *Nicotiana glauca* Graham, *Ricinus communis* L., *Tagetes erecta* L., *Taxus baccata* L., and *Thuja occidentalis* L. These plants, although endowed with undeniable decorative value, contain toxic secondary metabolites that can pose a serious risk in the event of accidental ingestion or contact, particularly for children and domestic animals [37,38,39]. In light of these considerations, it is essential to adopt a critical and selective approach in the choice of ornamental species to be introduced into new public and private gardens. The integration of ornamental and food plants can foster the creation of more resilient, multifunctional green spaces rooted in local traditions, while the exclusion or controlled management of toxic and allergenic species can help reduce risks to population health. In the long term, such strategies would not only enhance the horticultural heritage of Basilicata but also promote a model of ornamental greenery that is sustainable, safe, and aligned with community needs.

**Figure 7:**
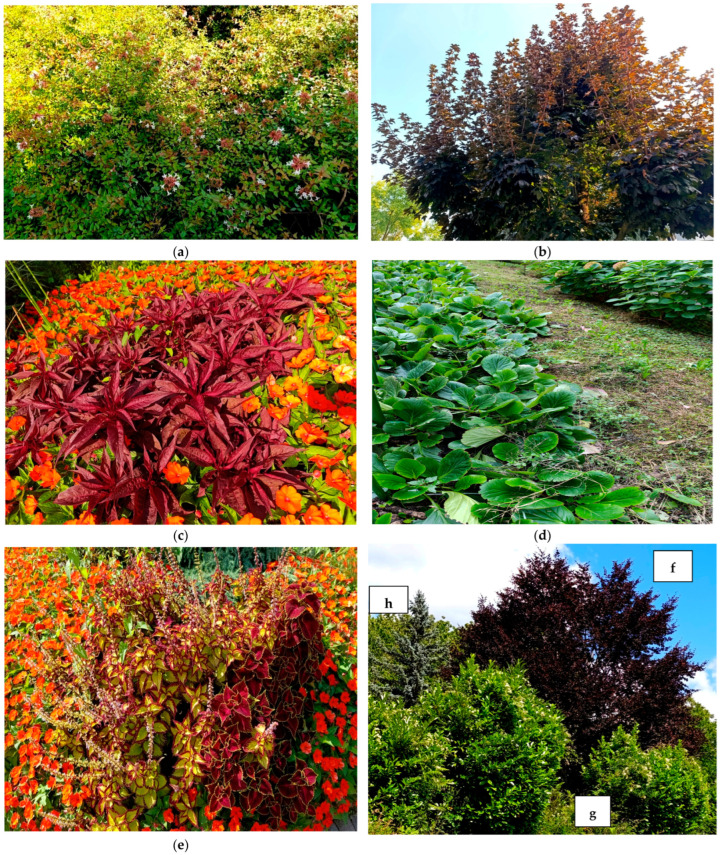

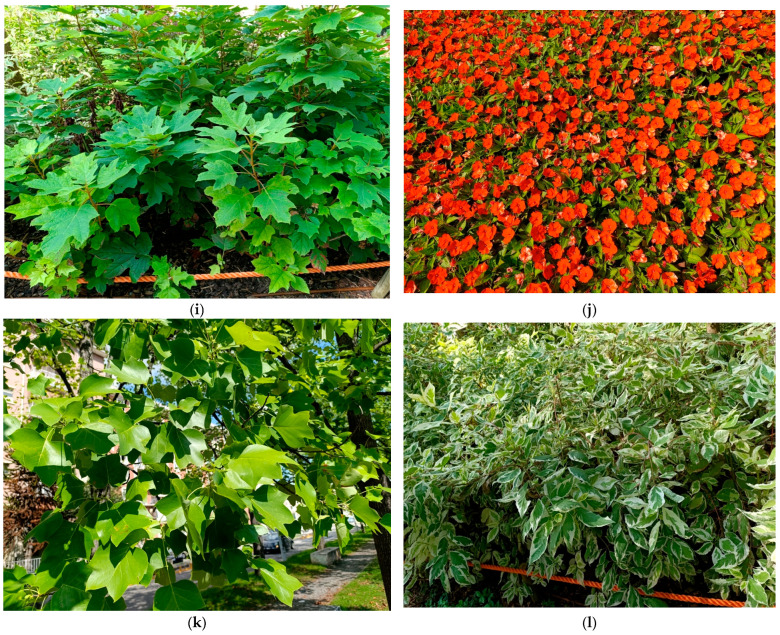
Ornamental species found in parks, public and private gardens, and along urban avenues in Basilicata. (**a**) *Abelia* × *grandiflora*, (**b**) *Acer pseudoplatanus* ‘Atropurpureum’, (**c**) *Amaranthus cruentus*, (**d**) *Bergenia crassifolia*, (**e**) *Coleus scutellarioides*, (**f**) *Fagus sylvatica* ‘Purpurea’, (**g**) *Viburnum odoratissimum*, (**h**) *Picea pungens* ‘Kosteriana’, (**i**) *Hydrangea quercifolia*, (**j**) *Impatiens hawkeri*, (**k**) *Liriodendron tulipifera*, and (**l**) *Weigela florida* ‘Variegata’.

Our investigation also revealed a considerable number of alien species in the flora of Basilicata, a phenomenon that is unfortunately widespread and observed worldwide. The predominant presence of alien ornamental taxa in the ornamental flora of Basilicata (almost 80%, of which 55% have begun the process of naturalization, with a large predominance of naturalized alien species) (Figure 6 and Figure 7) compared to native species represents an alert, as it shows how landscaping and ornamental choices of recent decades have favored the introduction of exotic species, often to the detriment of local plant components. This imbalance weakens the resilience of native flora, threatening local biodiversity and generating ecological imbalances that are difficult to contain. The massive use of alien ornamental plants becomes even more relevant when considering alien species that, in addition to competing with native flora, are dangerous to public health. This is the case, for example, of *Melia azedarach*, a plant of high ornamental value but characterized by high toxicity: its fruits are poisonous and potentially lethal, even if its young specimens are easily found in nurseries and the species is now naturalized in Basilicata. At the same time, the presence and spread of other invasive alien species pose a growing threat to local ecosystems. Some, such as *Acacia saligna* (Labill.) H. L. Wendl., *Ailanthus altissima*, *Carpobrotus acinaciformis* (L.) L. Bolus, *C. edulis* (L.) N.E.Br., *Opuntia ficus-indica* (L.) Mill., and *Robinia pseudoacacia* L., are known for their extraordinary ability to rapidly colonize new environments, altering natural habitats and denying native species of resources and space. All this makes these species particularly dangerous and difficult to manage, requiring interventions no longer limited to simple containment measures, but oriented towards true eradication programs. The eradication of invasive alien species is an action that, besides being complex and costly, is in some cases also difficult to implement. The eradication of *Ailanthus altissima*, for example, represents one of the most complex challenges in invasive species management. This plant, in fact, possesses an extraordinary vegetative regeneration capacity: even when cut or pollarded, it very rapidly produces a number of root suckers which not only ensure its survival but even favor its further spread. This characteristic makes simple mechanical interventions ineffective and often leads to an increase in population density, worsening the problem instead of containing it. For this reason, the management of tree-of-heaven requires integrated approaches that combine mechanical and chemical practices associated with repeated and long-term interventions, in order to truly reduce the vitality of the species and limit its spread [40,41].

In view of this, the problem of biological invasions can no longer be addressed as a marginal issue. Their spread, fueled by nursery practices not always attentive and by a growing demand for exotic ornamental species, today requires radically more effective prevention, awareness and management strategies. Recently, the issue of invasive species has finally returned to the center of the debate in Italy, with initiatives aimed not only at raising public awareness of the risks associated with their introduction and spread, but also at promoting good practices both in nursery production and gardening [23]. However, in order for such measures to produce concrete results, it will be necessary to complement education and prevention programs with constant commitment in terms of monitoring, timely eradication interventions, and stricter protection policies.

## 4. Materials and Methods

To assess the composition of ornamental plants cultivated in Basilicata, a survey was conducted in two main phases. First, data already available in the literature regarding the regional ornamental flora were examined [16,26].

Subsequently, between 2024 and 2025, direct field observations were carried out throughout the provinces of Basilicata. Specifically, surveys were conducted during the spring, summer, and autumn of 2024, and during the spring and summer of 2025, in order to observe plants during their flowering period and thus ensure accurate identification of the various taxa. In particular, we organized daily field excursions, and we excluded the ornamental plants cultivated inside botanical gardens and the surroundings of the biggest cities.

To represent the main climatic gradients of the region, the surveys included sites distributed along the coastal, hilly, and mountainous areas. In particular, urban and peri-urban environments were examined, including street tree plantings, historic villas, and both public and private gardens. The investigated localities cover 13 urban areas, which are reported in Table 1.

Taxonomic identification was performed with the support of authoritative floristic references [42,43,44,45,46,47,48,49,50]. Recorded taxa are listed in Appendix A Table A1. The nomenclature adopted follows the Plants of the World Online database (POWO, https://powo.science.kew.org, accessed on 5 September 2025) [27], and the taxon names are presented in alphabetical order. The checklist includes not only species sensu stricto but also infraspecific categories (subspecies, varieties, forms, cultivars) and hybrids. For each taxon, the following information was reported: family (according to [27]); growth form (according to [27,28]); geographic area of origin (derived from [27]); biome of origin (according to [27]); residence time (archaeophyte/neophyte) and status in Italy (native/alien), in accordance with [26,29].

## 5. Conclusions

Our investigation contributes to the knowledge of urban ornamental greenery, a field of research that has so far received limited attention at the national level but is gaining increasing interest, not only from a botanical perspective but also in practical and managerial terms. Urban ornamental greenery, in fact, represents a strategic element in territorial planning and in the sustainable management of public and private spaces, thereby requiring the development of targeted and informed strategies capable of integrating aesthetic, functional, and ecological requirements. In addition, our investigation contributes by initiating studies into the ornamental flora of Basilicata, which has remained largely unexplored until now. The number of taxa recorded, amounting to 281, is significant when compared to the limited size of the region, even though most of these are ornamental species commonly used in other Italian regions as well. The data collected in this first contribution provides a useful basis not only for further studies on the exotic component present in the Basilicata territory, but also to support municipal administrations in future actions of recovery, conservation, enhancement, and qualification of the plant heritage, with particular attention given to biodiversity protection and citizens’ well-being.

## Figures and Tables

**Table 1:** List of the main investigated localities.

City	Province	Localities	Geographical Coordinates
Potenza	Potenza	Villa del Prefetto	40°38′20″ N; 15°48′05″ E
		Villa di Santa Maria	40°38′37″ N; 15°48′15″ E
		Parco Baden Powell	40°38′58″ N; 15°47′51″ E
		Parco di Montereale	40°37′59″ N; 15°47′56″ E
		Parco del Fiore Bianco	40°39′27″ N; 15°48′22″ E
		Parco dell’Europa Unita	40°38′40″ N; 15°47′22″ E
		Parco fluviale del Basento	40°37′33″ N; 15°48′16″ E
		Parco Portofino	40°39′11″ N; 15°47′54″ E
		Parco del Seminario	40°38′08″ N; 15°48′09″ E
		Giardinetto di Parco Aurora	40°39′13″ N; 15°47′48″ E
		Corso G. Garibaldi	40°38′18″ N; 15°48′30″ E
		Corso Umberto I	40°38′12″ N; 15°48′08″ E
		Viale Dante	40°37′55″ N; 15°48′09″ E
		Via Baracca	40°38′07″ N; 15°48′32″ E
		Via D. di Giura	40°39′04″ N; 15°47′56″ E
		Rondò Tre Cancelli	40°39′02″ N; 15°48′01″ E
		Via E. Ciccotti	40°39′12″ N; 15°48′00″ E
		Via Siracusa	40°39′01″ N; 15°47′45″ E
		Viale Mediterraneo	40°38′04″ N; 15°47′18″ E
		P.zza Lattuchella	40°38′30″ N; 15°47′18″ E
Avigliano	Potenza	Villa del Monastero	40°43′56″ N; 15°43′13″ E
		Villa Falcone e Borsellino	40°43′47″ N; 15°43′16″ E
Campomaggiore Vecchio	Potenza	Parco dei Ruderi	40°34′35″ N; 16°05′50″ E
Maratea	Potenza	Villa Comunale Cardinale Gennari	39°59′49″ N; 15°43′35″ E
Melfi	Potenza	Villa Comunale	40°59′37″ N; 15°39′17″ E
Rionero in Vulture	Potenza	Villa Giulia Catena	40°55′22″N; 15°40′23″ E
		Villa Gen. Pennella	40°55′17″ N; 15°40′42″ E
		Giardino di Palazzo Fortunato	40°55′31″ N; 15°40′29″ E
Terranova di Pollino	Potenza	Via Convento	39°58′48″ N; 16°17′39″ E
Venosa	Potenza	Villa Comunale	40°57′37″ N; 15°48′50″ E
Matera	Matera	Villa Unità di Italia	40°40′10″ N; 16°36′24″ E
		Parco G. Paolo II	40°39′53″ N; 16°36′22″ E
		Parco Macamarda	40°40′04″ N; 16°35′49″ E
		Parco IV Novembre	40°40′16″ N; 16°36′00″ E
		Parco Rione Pini	40°39′27″ N; 16°36′42″ E
		Parco del Castello	40°39′50″ N; 16°36′23″ E
		Parco Serra Venerdì	40°40′09″ N; 16°35′24″ E
		Parco dei Quattro Evangelisti	40°40′32″ N; 16°34′40″ E
		Via Don Milani	40°39′35″ N; 16°36′35″ E
		Via Lanera	40°39′38″ N; 16°36′05″ E
		Via dei Dauni	40°40′59″ N; 16°34′55″ E
		Via dell′Agricoltura	40°40′31″ N; 16°34′24″ E
		Via Gravina	40°40′51″ N; 16°34′58″ E
		Via dei Bizantini	40°40′43″ N; 16°35′16″ E
		Via Lanfranchi	40°39′28″ N; 16°36′51″ E
Bernalda	Matera	Villa Comunale	40°24′28″ N; 16°41′12″ E
		Giardini di Palazzo Margherita	40°24′26″ N; 16°41′15″ E
Montescaglioso	Matera	Villa Belvedere Baden Powell	40°33′18″ N; 16°40′16″ E
Pisticci	Matera	Villa Comunale	40°23′17″ N; 16°33′39″ E
Policoro	Matera	Villa Comunale	40°12′35″ N; 16°40′39″ E
		Parco Angela Rocco	40°12′39″ N; 16°40′43″ E
		Giardini Murati	40°12′54″ N; 16°40′43″ E

## Data Availability

Data are contained within the article.

## Abbreviations

The following abbreviations are used in this manuscript:

POWO	Plants of the World Online database

## Appendix A

**Table A1:** List of ornamental taxa surveyed in the Basilicata region, sorted by family, growth form (according to [27,28]), area of geographical origin (derived from [27]), biome origin (according to [27]), time of residence (archaeophyte/neophyte) and status (native/alien) in Italy (both derived from [26,29]).

Family	Taxa	Growth Form	Geographical Origin	Biome Origin	Time of Residence	Status in Italy
Acanthaceae	*Acanthus mollis* L.	H scap	Mediterranean	Temperate		Native
Aizoaceae	*Carpobrotus acinaciformis* (L.) L.Bolus	Ch suffr	S Africa	Subtropical	Neophyte	Invasive alien
Aizoaceae	*Carpobrotus edulis* (L.) N.E.Br.	Ch suffr	S Africa	Subtropical	Neophyte	Invasive alien
Aizoaceae	*Mesembryanthemum cordifolium* L.f.	Ch suffr	Cape Province	Subtropical	Neophyte	Invasive alien
Altingiaceae	*Liquidambar styraciflua* L.	P scap	USA, C America	Temperate	Neophyte	Casual alien
Amaranthaceae	*Amaranthus cruentus* L.	T scap	S America	Tropical		Cultivated
Amaryllidaceae	*Agapanthus africanus* (L.) Hoffmanns.	G rhiz	S Africa	Subtropical		Cultivated
Amaryllidaceae	*Clivia miniata* Regel	G rhiz	S Africa	Subtropical		Cultivated
Amaryllidaceae	*Narcissus tazetta* L.	G bulb	Mediterranean	Subtropical		Native
Anacardiaceae	*Cotinus coggygria* Scop.	P caesp, P scap	C-S Europe, C Asia	Temperate		Native
Anacardiaceae	*Pistacia lentiscus* L.	P caesp, P scap	Mediterranean	Subtropical		Native
Anacardiaceae	*Schinus molle* L.	P scap	S America	Subtropical	Neophyte	Naturalized alien
Apocynaceae	*Carissa macrocarpa* (Eckl.) A.DC.	P caesp	S Africa	Shrubland	Neophyte	Naturalized alien
Apocynaceae	*Cascabela thevetia* (L.) Lippold	P caesp, P scap	C-S America	Tropical	Neophyte	Naturalized alien
Apocynaceae	*Catharanthus roseus* (L.) G.Don	Ch frut	Madagascar	Tropical	Neophyte	Naturalized alien
Apocynaceae	*Mandevilla laxa* (Ruiz & Pav.) Woodson	P lian	S America	Tropical		Cultivated
Apocynaceae	*Nerium oleander* L.	P caesp	Mediterranean, S-W Asia	Subtropical		Native
Apocynaceae	*Trachelospermum jasminoides* (Lindl.) Lem.	P lian	E Asia	Subtropical	Neophyte	Casual alien
Apocynaceae	*Vinca major* L.	Ch rept	S Europe, Caucasus	Temperate		Native
Apocynaceae	*Vinca major* var. variegata Loudon	Ch rept	Horticultural	Temperate		Cultivated
Aquifoliaceae	*Ilex aquifolium* L.	P caesp, P scap	Europe, N-W Africa	Temperate		Native
Aquifoliaceae	*Ilex aquifolium* L. ‘Aurea Marginata’	P scap	Horticultural	Temperate		Cultivated
Araceae	*Zantedeschia aethiopica* (L.) Spreng.	G rhiz	S Africa	Tropical	Neophyte	Invasive alien
Araliaceae	*Hedera canariensis* Willd.	P lian	Canarie Island	Temperate	Neophyte	Naturalized alien
Araliaceae	*Hedera helix* L.	P lian	Europe, W Asia	Temperate		Native
Araucariaceae	*Araucaria araucana* (Molina) K.Koch	P scap	S America	Temperate		Cultivated
Araucariaceae	*Araucaria columnaris* (G.Forst.) Hook.	P scap	New Caledonia	Tropical		Cultivated
Araucariaceae	*Araucaria heterophylla* (Salisb.) Franco	P scap	Norfolk Island	Tropical		Cultivated
Arecaceae	*Chamaerops humilis* L.	NP	Mediterranean	Subtropical		Native
Arecaceae	*Phoenix canariensis* H.Wildpret	P scap	Canarie Island	Subtropical	Neophyte	Naturalized alien
Arecaceae	*Syagrus romanzoffiana* (Cham.) Glassman	P scap	S America	Tropical		Casual alien
Arecaceae	*Trachycarpus fortunei* (Hook.) H.Wendl.	P scap	E Asia	Temperate	Neophyte	Invasive alien
Arecaceae	*Washingtonia filifera* var. robusta (H.Wendl.) Parish	P scap	Mexico	Shrubland	Neophyte	Naturalized alien
Asparagaceae	*Agave americana* L.	P caesp	Mexico	Tropical	Neophyte	Invasive alien
Asparagaceae	*Agave americana* var. marginata Trel.	P caesp	Mexico	Tropical	Neophyte	Invasive alien
Asparagaceae	*Asparagus falcatus* L.	P lian	S-E Africa, India	Tropical	Neophyte	Casual alien
Asparagaceae	*Asparagus setaceus* (Kunth) Jessop	G rhiz	S-E Africa	Tropical	Neophyte	Naturalized alien
Asparagaceae	*Aspidistra elatior* Blume	G rhiz	Japan	Subtropical	Neophyte	Casual alien
Asparagaceae	*Cordyline australis* (G.Forst.) Endl.	P caesp	New Zealand	Subtropical	Neophyte	Casual alien
Asparagaceae	*Ruscus aculeatus* L.	G rhiz	C-S Europe, N Africa, Caucasus	Temperate		Native
Asparagaceae	*Yucca gigantea* Lem.	P caesp	C America	Tropical	Neophyte	Casual alien
Asphodelaceae	*Aloe arborescens* Mill.	P succ	S Africa	Shrubland	Neophyte	Naturalized alien
Asphodelaceae	*Aloe vera* (L.) Burm.f.	P succ, NP	Oman	Shrubland	Archaeophyte	Naturalized alien
Asphodelaceae	*Kniphofia uvaria* (L.) Oken	Ch succ	Cape Province	Temperate	Neophyte	Casual alien
Asphodelaceae	*Phormium tenax* J.R.Forst. & G.Forst.	G rhiz	Norfolk Island, New Zealand	Temperate	Neophyte	Casual alien
Asphodelaceae	*Phormium tenax* J.R.Forst. & G.Forst. ‘Variegata’	G rhiz	Horticultural	Temperate		Cultivated
Asteraceae	*Calendula suffruticosa* Vahl	Ch suffr	S-W Mediterranean	Subtropical		Native
Asteraceae	*Chrysanthemum indicum* L.	Ch frut	E Asia	Temperate		Cultivated
Asteraceae	*Dahlia × hortensis* Guillaumin	G rhiz	Horticultural	Tropical		Cultivated
Asteraceae	*Dimorphotheca fruticosa* (L.) DC.	H scap	Cape Province	Subtropical	Neophyte	Casual alien
Asteraceae	*Dimorphotheca sinuata* DC.	H caesp	S Africa	Subtropical		Cultivated
Asteraceae	*Euryops pectinatus* (L.) Cass.	P caesp	S Africa	Subtropical		Cultivated
Asteraceae	*Farfugium japonicum* (L.) Kitam.	H caesp	China, Japan	Subtropical		Cultivated
Asteraceae	*Gazania rigens* (L.) Gaertn.	H caesp	S Africa	Subtropical	Neophyte	Naturalized alien
Asteraceae	*Helianthus annuus* L.	T scap	S America	Temperate	Neophyte	Casual alien
Asteraceae	*Helianthus tuberosus* L.	G bulb	N America	Temperate	Neophyte	Invasive alien
Asteraceae	*Senecio angulatus* L.f.	P lian	Cape Province	Subtropical	Neophyte	Invasive alien
Asteraceae	*Tagetes erecta* L.	T scap	C America	Subtropical	Neophyte	Casual alien
Asteraceae	*Zinnia elegans* Jacq.	T scap	C America	Tropical	Neophyte	Casual alien
Balsaminaceae	*Impatiens hawkeri* W.Bull	H scap	Papuasia	Tropical		Cultivated
Begoniaceae	*Begonia cucullata* Willd.	H scap	S America	Tropical		Cultivated
Berberidaceae	*Berberis thunbergii* DC. ‘Atropurpureum’	Ch suffr	Japan	Temperate		Cultivated
Berberidaceae	*Nandina domestica* Thunb.	P caesp	China	Temperate	Neophyte	Casual alien
Betulaceae	*Alnus cordata* Desf.	P scap	Corse	Temperate		Native
Betulaceae	*Corylus avellana* L.	P caesp	Europe, Caucasus	Temperate		Native
Betulaceae	*Ostrya carpinifolia* Scop.	P scap	S-C Europe, Caucasus	Temperate		Native
Bignoniaceae	*Campsis grandiflora* (Thunb.) K.Schum.	P lian	China, Japan	Temperate		Cultivated
Bignoniaceae	*Campsis radicans* (L.) Bureau	P lian	S-E USA	Subtropical	Neophyte	Naturalized alien
Bignoniaceae	*Catalpa bignonioides* Walter	P scap	N America	Temperate	Neophyte	Naturalized alien
Bignoniaceae	*Tecomaria capensis* (Thunb.) Spach	P caesp	S Africa	Subtropical	Neophyte	Casual alien
Buxaceae	*Buxus sempervirens* L.	NP	C-S Europe, N Africa, Caucasus	Temperate		Native
Cactaceae	*Opuntia ficus-indica* (L.) Mill.	P succ	Mexico	Tropical	Neophyte	Invasive alien
Cactaceae	*Opuntia humifusa* (Raf.) Raf.	P succ	USA, Mexico	Shrubland	Neophyte	Invasive alien
Cactaceae	*Opuntia tuna* (L.) Mill.	P succ	C America	Tropical	Neophyte	Naturalized alien
Calycanthaceae	*Calycanthus floridus* L.	P caesp	N America	Temperate	Neophyte	Casual alien
Cannabaceae	*Celtis australis* L.	P scap	S Europe, N-W Africa, Caucasus	Subtropical		Native
Cannaceae	*Canna indica* L.	G rhiz	C-S America	Tropical	Neophyte	Naturalized alien
Capparaceae	*Capparis spinosa* L.	NP	Mediterranean	Subtropical		Native
Caprifoliaceae	*Abelia × grandiflora* (Rovelli ex André) Rehder	P caesp	Artificial hybrid	Temperate		Cultivated
Caprifoliaceae	*Lonicera japonica* Thunb.	P caesp	E Asia	Temperate	Neophyte	Invasive alien
Caprifoliaceae	*Symphoricarpos albus* (L.) S.F.Blake	P caesp	N America, Mexico	Temperate	Neophyte	Naturalized alien
Caprifoliaceae	*Weigela florida* (Bunge) A.DC. ‘Variegata’	P caesp	Horticultural	Temperate		Cultivated
Celastraceae	*Euonymus japonicus* Thunb.	P caesp	Japan	Subtropical	Neophyte	Naturalized alien
Celastraceae	*Euonymus japonicus* Thunb. ‘Aureomarginatus’	P caesp	Japan	Subtropical		Cultivated
Commelinaceae	*Tradescantia fluminensis* Vell.	G rhiz	S America	Tropical	Neophyte	Invasive alien
Commelinaceae	*Tradescantia pallida* (Rose) D.R.Hunt	G rhiz	Mexico	Tropical	Neophyte	Casual alien
Convolvulaceae	*Ipomoea purpurea* (L.) Roth	P lian	C-S America	Tropical	Neophyte	Naturalized alien
Crassulaceae	*Aeonium arboreum* (L.) Webb & Berthel.	NP	Canarie Island	Subtropical	Archaeophyte	Invasive alien
Crassulaceae	*Aeonium arboreum* (L.) Webb & Berthel. ‘Atropurpureum’	NP	Canarie Island	Subtropical		Cultivated
Crassulaceae	*Crassula ovata* (Mill.) Druce	NP succ	S Africa	Subtropical	Neophyte	Casual alien
Crassulaceae	*Petrosedum rupestre* (L.) P.V.Heath	Ch succ	C-W Europe, Turkey	Temperate		Native
Cupressaceae	*Chamaecyparis lawsoniana* (A.Murray bis) Parl.	P scap	N America	Temperate	Neophyte	Casual alien
Cupressaceae	*Cupressus sempervirens* f. horizontalis (Mill.) Voss	P scap	Mediterranean, Iran	Temperate	Archaeophyte	Naturalized alien
Cupressaceae	*Cupressus sempervirens* L.	P scap	Mediterranean, Iran	Temperate	Archaeophyte	Naturalized alien
Cupressaceae	*Hesperocyparis arizonica* (Greene) Bartel	P scap	N America	Temperate	Neophyte	Naturalized alien
Cupressaceae	*Hesperocyparis macrocarpa* (Hartw.) Bartel	P scap	California	Temperate	Neophyte	Naturalized alien
Cupressaceae	×*Hesperotropsis leylandii* (A.B.Jacks. & Dallim.) Garland & Gerry Moore	P scap	Artificial hybrid	Temperate		Cultivated
Cupressaceae	*Juniperus chinensis* f. pfltzeriana (Spaeth) Rehder	P caesp, P scap	E Asia	Temperate	Neophyte	Casual alien
Cupressaceae	*Platycladus orientalis* (L.) Franco	P scap	C-E Asia	Temperate	Neophyte	Naturalized alien
Cupressaceae	*Sequoia sempervirens* (D.Don) Endl.	P scap	Oregon, California,	Temperate	Neophyte	Naturalized alien
Cupressaceae	*Thuja occidentalis* L.	P scap	N-E America	Temperate	Neophyte	Casual alien
Cupressaceae	*Thuja plicata* Donn ex D.Don	P scap	N America	Temperate	Neophyte	Casual alien
Cycadaceae	*Cycas revoluta* Thunb.	P caesp	China, Japan, Taiwan	Subtropical	Neophyte	Casual alien
Cyperaceae	*Cyperus alternifolius* L.	G rhiz	Madagascar	Tropical	Neophyte	Invasive alien
Ebenaceae	*Diospyros kaki* Thunb.	P scap	E Asia	Temperate	Neophyte	Naturalized alien
Elaeagnaceae	*Elaeagnus angustifolia* L.	P scap	C Asia	Temperate	Neophyte	Naturalized alien
Elaeagnaceae	*Elaeagnus pungens* Thunb.	P scap	China	Temperate	Neophyte	Invasive alien
Elaeagnaceae	*Elaeagnus × submacrophylla* Servett.	P scap	Korea, Japan	Temperate	Neophyte	Casual alien
Ericaceae	*Arbutus unedo* L.	P scap	Mediterranean	Temperate		Native
Ericaceae	*Rhododendron arboreum* Sm.	P caesp	India, Tibet	Temperate		Cultivated
Ericaceae	*Rhododendron simsii* Planch.	P caesp	China, Taiwan	Subtropical		Cultivated
Ericaceae	*Rhododendron japonicum* (A.Gray) Suringar	P caesp	Japan	Temperate		Cultivated
Euphorbiaceae	*Euphorbia pulcherrima* Willd.	NP	C America	Tropical	Neophyte	Casual alien
Euphorbiaceae	*Ricinus communis* L.	P caesp, P scap	N-E Africa	Tropical	Archaeophyte	Invasive alien
Fabaceae	*Acacia dealbata* Link	P scap	Australia	Temperate	Neophyte	Invasive alien
Fabaceae	*Acacia saligna* (Labill.) H.L.Wendl.	P scap	Australia	Subtropical	Neophyte	Invasive alien
Fabaceae	*Albizia julibrissin* Durazz.	P scap	S Asia	Temperate	Neophyte	Casual alien
Fabaceae	*Ceratonia siliqua* L.	P scap	Mediterranean, Caucasus	Subtropical		Native
Fabaceae	*Cercis siliquastrum* L.	P scap	S-E Europe	Temperate		Native
Fabaceae	*Erythrostemon gilliesii* (Hook.) Klotzsch	P scap	S America	Temperate	Neophyte	Naturalized alien
Fabaceae	*Laburnum anagyroides* Medik.	P caesp	S Europe	Temperate		Native
Fabaceae	*Robinia pseudoacacia* L.	P scap	N America	Temperate	Neophyte	Invasive alien
Fabaceae	*Spartium junceum* L.	P caesp	Mediterranean	Temperate		Native
Fabaceae	*Wisteria sinensis* (Sims) DC.	P lian	China	Temperate	Neophyte	Naturalized alien
Fagaceae	*Castanea sativa* Mill.	P scap	Balkans, Caucasus	Temperate		Native
Fagaceae	*Fagus sylvatica* L. ‘Purpurea’	P scap	Horticultural	Temperate		Cultivated
Fagaceae	*Quercus cerris* L.	P scap	C-S Europe, Caucasus	Temperate		Native
Fagaceae	*Quercus ilex* L.	P scap	S Europe, Mediterranean	Temperate		Native
Fagaceae	*Quercus petraea* (Matt.) Liebl.	P scap	Europe, Caucasus	Temperate		Native
Fagaceae	*Quercus pubescens* Willd. s.l.	P scap	C-W Mediterranean	Temperate		Cultivated
Fagaceae	*Quercus robur* L.	P scap	Europe, Caucasus	Temperate		Native
Fagaceae	*Quercus suber* L.	P scap	C-W Mediterranean	Temperate		Native
Garryaceae	*Aucuba japonica* Thunb.	P caesp	E Asia	Temperate	Neophyte	Casual alien
Geraniaceae	*Pelargonium peltatum* (L.) L’Hér.	Ch suffr	Cape Province	Subtropical	Neophyte	Casual alien
Geraniaceae	*Pelargonium zonale* (L.) L’Hér.	Ch suffr	Cape Province	Subtropical		Cultivated
Ginkgoaceae	*Ginkgo biloba* L.	P scap	China	Temperate	Neophyte	Casual alien
Hamamelidaceae	*Loropetalum chinense* (R.Br.) Oliv.	P caesp	China, Japan	Temperate		Cultivated
Hyacinthaceae	*Hyacinthus orientalis* L.	G bulb	W Asia	Temperate	Archaeophyte	Casual alien
Hydrangeaceae	*Deutzia scabra* Thunb.	P caesp	Japan	Temperate		Cultivated
Hydrangeaceae	*Hydrangea macrophylla* (Thunb.) Ser.	P caesp	Japan	Temperate	Neophyte	Naturalized alien
Hydrangeaceae	*Hydrangea quercifolia* W. Bartram	P caesp	USA	Temperate		Cultivated
Hydrangeaceae	*Philadelphus coronarius* L.	P caesp	Caucasus	Temperate		Native
Iridaceae	*Freesia refracta* (Jacq.) Klatt	G bulb	S Africa	Subtropical		Cultivated
Iridaceae	*Freesia leichtlinii* Klatt subsp. *alba* (G.L.Mey.) J.C.Manning & Goldblatt	G bulb	Cape Province	Subtropical	Neophyte	Naturalized alien
Iridaceae	*Iris × germanica* L.	G rhiz	E Mediterranean	Temperate	Archaeophyte	Naturalized alien
Juglandaceae	*Juglans regia* L.	P scap	Caucasus	Temperate		Cryptogenic
Lamiaceae	*Coleus scutellarioides* (L.) Benth.	H scap	S-E Asia, Australia	Tropical	Neophyte	Casual alien
Lamiaceae	*Lavandula angustifolia* Mill.	NP	S-W Europe	Temperate		Native
Lamiaceae	*Salvia offiChinalis* L.	Ch suffr	C-S Europe	Temperate		Native
Lamiaceae	*Salvia rosmarinus* Spenn.	NP	Mediterranean	Temperate		Native
Lamiaceae	*Vitex agnus-castus* L.	P caesp, P scap	Mediterranean, C-S Asia	Subtropical		Native
Lauraceae	*Laurus nobilis* L.	P caesp, P scap	Mediterranean	Subtropical		Native
Liliaceae	*Lilium candidum* L.	G bulb	E Mediterranean, W Asia	Temperate	Archaeophyte	Naturalized alien
Liliaceae	*Tulipa agenensis* Redouté	G bulb	E Mediterranean	Subtropical	Neophyte	Naturalized alien
Liliaceae	*Tulipa raddii* Reboul	G bulb	Turkey	Temperate	Neophyte	Naturalized alien
Lythraceae	*Cuphea hyssopifolia* Kunth	Ch suffr	C America	Tropical	Neophyte	Casual alien
Lythraceae	*Lagerstroemia indica* L.	P caesp, P scap	S Asia	Subtropical	Neophyte	Casual alien
Lythraceae	*Punica granatum* L.	P scap	Caucasus	Temperate	Archaeophyte	Naturalized alien
Magnoliaceae	*Liriodendron tulipifera* L.	P scap	N America	Temperate	Neophyte	Naturalized alien
Magnoliaceae	*Magnolia grandiflora* L.	P scap	N America	Subtropical	Neophyte	Casual alien
Magnoliaceae	*Magnolia × soulangeana* Soul.-Bod.	P scap	China	Temperate		Cultivated
Malvaceae	*Abutilon theophrasti* Medik.	P caesp	C Asia	Subtropical	Archaeophyte	Invasive alien
Malvaceae	*Hibiscus syriacus* L.	P caesp	E Asia	Temperate		Casual alien
Malvaceae	*Hibiscus × rosa-sinensis* L.	P caesp	W Pacific	Tropical	Neophyte	Casual alien
Malvaceae	*Tilia cordata* Mill.	P scap	Europe, N-C Asia	Temperate		Native
Malvaceae	*Tilia platyphyllos* Scop.	P scap	Europe, Caucasus	Temperate		Native
Meliaceae	*Melia azedarach* L.	P scap	E Asia, Australia	Tropical	Neophyte	Naturalized alien
Moraceae	*Broussonetia papyrifera* (L.) Vent.	P scap	E Asia	Temperate	Neophyte	Invasive alien
Moraceae	*Morus alba* L.	P scap	E Asia	Temperate	Archaeophyte	Naturalized alien
Moraceae	*Morus nigra* L.	P scap	W Asia	Temperate	Archaeophyte	Naturalized alien
Musaceae	*Musa × paradisiaca* L.	G rhiz	Malaysia	Tropical	Neophyte	Casual alien
Myrtaceae	*Eucalyptus camaldulensis* Dehnh.	P scap	Australia	Shrubland	Neophyte	Invasive alien
Myrtaceae	*Metrosideros excelsa* Sol. ex Gaertn.	P scap	New Zealand	Subtropical	Neophyte	Casual alien
Myrtaceae	*Myrtus communis* L.	P caesp, P scap	Mediterranean, W Asia	Temperate		Native
Myrtaceae	*Myrtus communis* subsp. tarentina (L.) Nyman	P caesp, P scap	S Europe	Temperate		Native
Nyctaginaceae	*Bougainvillea glabra* Choisy	P lian	C-S America	Tropical	Neophyte	Casual alien
Nyctaginaceae	*Bougainvillea spectabilis* Willd.	P lian	C-S America	Tropical	Neophyte	Casual alien
Nyctaginaceae	*Mirabilis jalapa* L.	G bulb	C America	Subtropical	Neophyte	Invasive alien
Nymphaeaceae	*Nymphaea alba* L.	I rad	Europe, N Africa, N-C Asia	Temperate		Native
Oleaceae	*Chrysojasminum fruticans* (L.) Banfi	P caesp	Mediterranean, Caucasus	Temperate		Native
Oleaceae	*Forsythia × intermedia* Zabel	P scap	Artificial hybrid	Subtropical	Neophyte	Casual alien
Oleaceae	*Fraxinus angustifolia* Vahl	P scap	Mediterranean	Temperate		Native
Oleaceae	*Fraxinus ornus* L.	P scap	Mediterranean	Temperate		Native
Oleaceae	*Jasminum polyanthum* Franch.	P caesp	China	Subtropical	Neophyte	Casual alien
Oleaceae	*Ligustrum japonicum* Thunb.	P caesp, P scap	E Asia	Temperate	Neophyte	Casual alien
Oleaceae	*Ligustrum lucidum* W.T.Aiton	P scap	E Asia	Temperate	Neophyte	Invasive alien
Oleaceae	*Ligustrum lucidum* W.T.Aiton ‘Excelsum Superbum’	P scap	Horticultural	Temperate		Cultivated
Oleaceae	*Ligustrum ovalifolium* Hassk.	P caesp, P scap	Japan	Temperate	Neophyte	Invasive alien
Oleaceae	*Ligustrum ovalifolium* Hassk. ‘Aureum’	P caesp, P scap	Japan	Temperate		Cultivated
Oleaceae	*Ligustrum sinense* Lour.	P caesp, P scap	S-E Asia	Subtropical	Neophyte	Invasive alien
Oleaceae	*Ligustrum vulgare* L.	P caesp	Europe, N Africa, Iran	Temperate		Native
Oleaceae	*Olea Europeea* L.	P scap	Mediterranean, Africa, S-W Asia	Temperate		Native
Oleaceae	*Osmanthus fragrans* Lour.	P caesp, P scap	C-E Asia	Subtropical		Cultivated
Oleaceae	*Syringa vulgaris* L.	P caesp, P scap	S-E Europe	Temperate	Neophyte	Naturalized alien
Onagraceae	*Fuchsia × standishii* J.Harrison	P scap	Artificial hybrid	Tropical		Cultivated
Paeoniaceae	*Paeonia × suffruticosa* Andrews	Ch suffr	China	Temperate	Neophyte	Historical record
Passifloraceae	*Passiflora caerulea* L.	P lian	S America	Subtropical	Neophyte	Naturalized alien
Paulowniaceae	*Paulownia tomentosa* (Thunb.) Steud.	P scap	China, Korea	Temperate	Neophyte	Invasive alien
Phytolaccaceae	*Phytolacca americana* L.	P scap	N-C America	Temperate	Neophyte	Invasive alien
Pinaceae	*Abies alba* Mill.	P scap	S Europe	Temperate		Native
Pinaceae	*Abies cephalonica* Loudon	P scap	Greece	Temperate	Neophyte	Invasive alien
Pinaceae	*Abies nordmanniana* (Steven) Spach	P scap	Caucasus	Temperate		Cultivated
Pinaceae	*Abies pinsapo* Boiss.	P scap	Spain	Temperate		Cultivated
Pinaceae	*Cedrus atlantica* (Endl.) Manetti ex Carrière	P scap	N-W Africa	Temperate	Neophyte	Naturalized alien
Pinaceae	*Cedrus deodara* (Roxb. ex D.Don) G.Don	P scap	C Asia	Temperate	Neophyte	Naturalized alien
Pinaceae	*Cedrus libani* A.Rich.	P scap	Turkey, Lebanon	Temperate	Neophyte	Casual alien
Pinaceae	*Picea abies* (L.) H.Karst.	P scap	Europe, N-W Asia	Temperate		Native
Pinaceae	*Picea orientalis* (L.) Peterm.	P scap	Turkey/Caucasus	Temperate		Cultivated
Pinaceae	*Picea pungens* Engelm. ‘Kosteriana’	P scap	N America	Temperate		Cultivated
Pinaceae	*Pinus halepensis* Mill.	P scap	Mediterranean	Temperate		Native
Pinaceae	*Pinus nigra* J.F.Arnold	P scap	S-E Europe, Caucasus	Temperate		Native
Pinaceae	*Pinus pinea* L.	P scap	S Europe, Lebanon	Temperate	Archaeophyte	Naturalized alien
Pittosporaceae	*Pittosporum tobira* (Thunb.) W.T.Aiton	P caesp	Korea, Japan	Subtropical	Neophyte	Naturalized alien
Pittosporaceae	*Pittosporum tobira* (Thunb.) W.T.Aiton ‘Albomarginata’	P caesp	Korea, Japan	Subtropical		Cultivated
Pittosporaceae	*Pittosporum tobira* (Thunb.) W.T.Aiton ‘Nanum’	P caesp	Horticultural	Subtropical		Cultivated
Plantaginaceae	*Antirrhinum majus* L.	Ch frut	Spain, France	Temperate	Archaeophyte	Naturalized alien
Platanaceae	*Platanus × hispanica* Mill. ex Münchh.	P scap	Artificial hybrid	Temperate	Neophyte	Invasive alien
Plumbaginaceae	*Plumbago auriculata* Lam.	P lian	S Africa	Subtropical	Neophyte	Naturalized alien
Poaceae	*Bambusa vulgaris* Schrad. ex J.C.Wendl.	P scap	China	Tropical		Cultivated
Poaceae	*Cortaderia selloana* (Schult. & Schult.f.) Asch. & Graebn.	H caesp	S America	Subtropical	Neophyte	Invasive alien
Poaceae	*Phyllostachys nigra* (Lodd. ex Lindl.) Munro	P caesp	China	Temperate	Neophyte	Naturalized alien
Polygonaceae	*Polygala myrtifolia* L.	NP	S Africa	Subtropical	Neophyte	Casual alien
Portulacaceae	*Portulaca grandiflora* Hook.	T scap	S America	Subtropical	Neophyte	Naturalized alien
Primulaceae	*Cyclamen persicum* Mill.	G bulb	Algeria, E Mediterranean	Subtropical	Neophyte	Naturalized alien
Pteridaceae	*Adiantum capillus-veneris* L.	G rhiz	Cosmopolitan	Temperate		Native
Ranunculaceae	*Clematis × jackmanii* T.Moore	P lian	Artificial hybrid	Temperate		Cultivated
Rhamnaceae	*Rhamnus alaternus* L.	P caesp	Mediterranean	Temperate		Native
Rosaceae	*Chaenomeles speciosa* (Sweet) Nakai	P scap	China	Temperate	Neophyte	Casual alien
Rosaceae	*Cotoneaster pannosus* Franch.	P caesp	China	Temperate	Neophyte	Naturalized alien
Rosaceae	*Crataegus monogyna* Jacq.	P caesp	Europe, N-W Africa, Caucasus	Temperate		Native
Rosaceae	*Cydonia oblonga* Mill.	P scap	S-W Asia	Temperate	Archaeophyte	Naturalized alien
Rosaceae	*Eriobotrya japonica* (Thunb.) Lindl.	P scap	China	Temperate	Neophyte	Naturalized alien
Rosaceae	*Kerria japonica* (L.) DC.	P caesp	China, Japan	Temperate	Neophyte	Naturalized alien
Rosaceae	*Photinia serratifolia* (Desf.) Kalkman	P caesp, P scap	E Asia	Temperate	Neophyte	Casual alien
Rosaceae	*Photinia × fraseri* Dress	P caesp, P scap	China	Temperate		Cultivated
Rosaceae	*Prunus cerasifera* Ehrh. ‘Pissardi’	P scap	Horticultural	Temperate		Naturalized alien
Rosaceae	*Prunus laurocerasus* L.	P scap	S-E Europe, Caucasus	Temperate	Neophyte	Invasive alien
Rosaceae	*Prunus serrulata* Lindl.	P scap	E Asia	Temperate		Cultivated
Rosaceae	*Pyracantha coccinea* M.Roem.	P caesp	C-S Europe, Caucasus	Temperate		Native
Rosaceae	*Rosa banksiae* W.T.Aiton	NP	C hina	Temperate	Neophyte	Naturalized alien
Rosaceae	*Rosa × centifolia* L.	NP	Artificial hybrid	Temperate	Archaeophyte	Casual alien
Rosaceae	*Rosa* × *hybrida* Vill.	NP	Artificial hybrid	Temperate		Cultivated
Rosaceae	*Spiraea × vanhouttei* (Briot) Carrière	P caesp	Artificial hybrid	Temperate	Neophyte	Casual alien
Rutaceae	*Citrus × aurantium* L.	P scap	S-E Asia	Subtropical	Archaeophyte	Casual alien
Rutaceae	*Citrus × limon* (L.) Osbeck	P scap	Artificial hybrid	Subtropical	Archaeophyte	Casual alien
Salicaceae	*Populus alba* L.	P scap	C-S Europe, C Asia	Temperate		Native
Salicaceae	*Populus nigra* L.	P scap	Europe, N Africa, O Asia	Temperate		Native
Salicaceae	*Salix babylonica* L.	P scap	China, Korea	Temperate	Neophyte	Casual alien
Sapindaceae	*Acer campestre* L.	P scap	Europe, N-W Africa, W Asia	Temperate		Native
Sapindaceae	*Acer negundo* L.	P scap	N-C America	Temperate	Neophyte	Invasive alien
Sapindaceae	*Acer palmatum* Thunb. ‘Atropurpureum’	P scap	Horticultural	Temperate		Cultivated
Sapindaceae	*Acer pseudoplatanus* L.	P scap	Europe, Caucasus	Temperate		Native
Sapindaceae	*Acer pseudoplatanus* L. ‘Atropurpureum’	P scap	Horticultural	Temperate		Cultivated
Sapindaceae	*Aesculus hippocastanum* L.	P scap	Balkans, Turkey	Temperate	Neophyte	Invasive alien
Saxifragaceae	*Bergenia crassifolia* (L.) Fritsch	Ch caesp	C-N Asia	Temperate	Neophyte	Casual alien
Scrophulariaceae	*Buddleja davidii* Franch.	P caesp	China	Temperate	Neophyte	Invasive alien
Simaroubaceae	*Ailanthus altissima* (Mill.) Swingle	P scap	China	Temperate	Neophyte	Invasive alien
Solanaceae	*Brugmansia arborea* (L.) Sweet	P scap	C-S America	Tropical		Cultivated
Solanaceae	*Capsicum annuum* L.	T scap	S America	Tropical	Neophyte	Casual alien
Solanaceae	*Cestrum nocturnum* L.	P caesp	C-S America	Tropical		Cultivated
Solanaceae	*Cestrum parqui* (Lam.) L’Hér.	P caesp	S America	Subtropical	Neophyte	Invasive alien
Solanaceae	*Nicotiana glauca* Graham	P caesp	Bolivia, Brazil	Subtropical	Neophyte	Invasive alien
Solanaceae	*Petunia × atkinsiana* (Sweet) D.Don ex W.H.Baxter	T scap	Artificial hybrid	Subtropical	Neophyte	Naturalized alien
Strelitzaceae	*Strelitzia reginae* Banks	G rhiz	Cape Province	Subtropical		Cultivated
Tamaricaceae	*Tamarix africana* Poir.	P caesp, P scap	W Mediterranean	Subtropical	Neophyte	Native
Tamaricaceae	*Tamarix arborea* (Sieber ex Ehrenb.) Bunge	P scap	N Africa, S Europe	Shrubland		Native
Tamaricaceae	*Tamarix canariensis* Willd.	P caesp	Canarie Island	Subtropical		Cultivated
Tamaricaceae	*Tamarix gallica* L.	P caesp	S-W Europe, N-W Africa	Temperate		Native
Taxaceae	*Taxus baccata* L.	P scap	Europe, N-W Africa, Caucasus	Temperate		Native
Taxaceae	*Taxus baccata* L. ‘Fastigiata’	P scap	Horticultural	Temperate		Cultivated
Taxaceae	*Taxus baccata* L. ‘Fastigiata Aurea’	P scap	Horticultural	Temperate		Cultivated
Theaceae	*Camellia japonica* L.	P scap	S-E Asia	Subtropical		Cultivated
Ulmaceae	*Ulmus minor* subsp. canescens Bartolucci & Galasso	P scap	C-W-E Mediterranean	Temperate		Native
Verbenaceae	*Duranta erecta* L.	P caesp	C-S America	Tropical		Cultivated
Verbenaceae	*Lantana camara* L.	P caesp	C-S America	Subtropical	Neophyte	Naturalized alien
Viburnaceae	*Viburnum odoratissimum* Ker Gawl.	P caesp	Asia	Subtropical		Cultivated
Viburnaceae	*Viburnum opulus* L.	P caesp	Europe, N-C Asia, N-W Africa	Temperate		Native
Viburnaceae	*Viburnum rhytidophyllum* Hemsl.	P caesp, P scap	China	Temperate	Neophyte	Casual alien
Viburnaceae	*Viburnum tinus* L.	P caesp	Mediterranean	Subtropical		Native
Vitaceae	*Parthenocissus quinquefolia* (L.) Planch.	P lian	N America	Temperate	Neophyte	Invasive alien
Vitaceae	*Parthenocissus tricuspidata* (Siebold & Zucc.) Planch.	P lian	E Asia	Temperate	Neophyte	Naturalized alien
